# Cancer-related pain in long-term survivors of oncological diseases: results of a survey on the current care situation

**DOI:** 10.1007/s00520-024-09081-2

**Published:** 2024-12-20

**Authors:** H. Hofbauer, K. Kieselbach, S. Wirz, A. Bundscherer, U. M. Stamer, F. Rapp

**Affiliations:** 1 Pain Therapy Unit, Department of Anaesthesiology and Intensive Care Medicine, University Hospital Ulm, Albert-Einstein-Allee 23, 89075 Ulm, Germany; 2 Interdisciplinary Pain Center, Medical Center University Freiburg, Breisacher Str. 117, 79016 Freiburg, Germany; 3 Department for Anaesthesiology, Intensive Medicine, Pain and Palliative Medicine, Centre for Pain Medicine, Weaning Center, University of Bonn, Cura Hospital/GFO-Clinics Bonn, Schuelgenstr. 15, 53604 Bad Honnef, Germany; 4 Department of Anaesthesiology, University Medical Center of Regensburg, Regensburg, Germany; 5 Department of Anaesthesiology and Pain Medicine, Inselspital, Bern University Hospital, University of Bern, Freiburgstrasse, 3010 Bern, Switzerland

**Keywords:** Cancer-related pain, Pain chronification, Long-term survivors, Interdisciplinary multimodal pain therapy, Healthcare research

## Abstract

**Purpose:**

The increasing survival rates of oncology patients have led to a corresponding increase in long-time survivors living with chronic cancer-related pain. Data is scarce on the care situation for this distinct clinical entity and on specific therapy requirements, such as interdisciplinary, multimodal pain therapy (IMPT). Our cross-sectional study aimed to assess the current care situation, distinct chronification factors, and optimization potential. This survey addresses this need in Germany, but also provides results with international implications.

**Methods:**

Via an online survey, German Pain Society members involved in the treatment of long-time survivors with chronic cancer-related pain assessed the current care situation, chronification factors, specific treatment needs, and the required practitioner’s expertise. The German Pain Society’s Cancer Pain Working Group created the non-validated questionnaire using the Delphi method.

**Results:**

One hundred fifty-nine Pain Society members across 70% of Germany’s postal regions answered our survey. Respondents (primarily physicians, and 75% with + 6 years of experience) assessed the care situation as worse for chronic cancer-related pain compared to acute pain. Only 10% of the sites provided specific therapy for chronic cancer-related pain (mostly via outpatient treatment). Compared to non-cancer-related pain, additional, cancer-specific chronification factors were assumed, especially at psychological levels, and these need incorporating into therapies. A majority of practitioners recommended cancer-specific IMPT and specific pain expertise for this distinct clinical entity.

**Conclusions:**

Members from the German Pain Society assume that there are relevant deficits in the care of long-term survivors with chronic cancer-related pain. The situation may be assessed differently by other groups, e.g., oncologists, and the data relates to Germany. Nevertheless, considering the raising survival rates, it can be supposed that there is reason to be concerned about an increasing care deficit. Thus, besides expanding the range of available treatment and raising awareness, IMPT with specially trained personnel should be developed to address the care needs of cancer survivors experiencing chronic cancer-related pain.

**Supplementary Information:**

The online version contains supplementary material available at 10.1007/s00520-024-09081-2.

## Introduction

The annual incidence of oncological diseases in Germany is around 500,000 and survival rates have continuously improved in recent years [1–3]. According to Germany’s Federal Ministry of Health, there are likely 2.6 million long-term survivors whose first cancer diagnosis was at least 5 years ago, regardless of their current cancer status, including those in a stable palliative situation [4]. Given the improved survival chances, awareness is also increasing about protracted disease trajectories and the associated complications such as long-term side effects. In the USA, cancer survivors comprised around 5% of the total population in January 2019. By 2030, this figure is expected to rise by around 30% to more than 22 million long-term survivors [5]. According to a meta-analysis, the overall prevalence of persistent cancer-related pain in patients post-therapy is 35.8% with a prevalence of moderate to severe pain of 22.8% [6], which represents a high psychosocial burden and challenge for patients, their relatives, and healthcare professionals. In Germany, this corresponds to around 0.5 to 1 million long-term survivors suffering from chronic cancer-related pain.

Mechanisms for the chronification of cancer-related pain are similar to those for non-cancer pain [7]. There are also tumor-specific causes, including the release of pain-triggering inflammatory mediators, and psychological factors such as fear of recurrence [8], which are reflected in increased pain impairment [9–11].

Cancer-related pain is still mostly treated pharmacologically according to the WHO ladder, including opioids. This therapeutic approach often reaches its limits with increasing pain chronification and pain-related impairment, as it does not take sufficient account of the implications of the biopsychosocial pain model. However, there are scarcely any services specifically tailored to chronic cancer-related pain. Interdisciplinary, multimodal pain therapy (IMPT) is a recommended comprehensive treatment method for chronic pain management in line with the biopsychosocial pain model to improve strategies for dealing with chronic pain [12, 13]. The biopsychosocial disease model identifies pain chronification and pain perpetuation factors. These can meet the criteria for chronic pain disorder in the sense of a disease in its own right [14]. After a careful pain assessment, an indication for IMPT can be determined. IMPT is a “closely coordinated, interdisciplinary, team-integrated treatment in small groups involving somatic, physical, and psychological exercise and psychotherapeutic procedures” [12]. Strong evidence speaks to recommending IMPT for effectively managing chronic pain disorders effectively [12, 15]. Effective interdisciplinary pain therapy requires both a mechanism-based and individualized approach. In contrast, similar more specified treatment recommendations for chronic cancer-related pain do not exist [16], which means that the care situation for this patient group is currently unclear.

Therefore, our cross-sectional study aimed to gain clarity by surveying members of an organization with particular expertise in pain therapy, the German Pain Society (Deutsche Schmerzgesellschaft e.V.). Via an online survey, we asked Society members to first assess the current care situation in Germany for patients with chronic cancer-related pain. Then, we queried about optimization potential in the treatment of long-term survivors with chronic pain so as to contribute to better care for this patient group in the future.

## Methods

### Study design and procedure

We sent per e-mail an anonymized online survey (Unipark software EFS Spring 2023 Tivian XI GmbH, Cologne) created by the German Pain Society’s Cancer Pain Working Group to all Society members. However, we emphasized our interest in having experts who treat patients with cancer-related pain complete the survey. Using a link or QR code, recipients could access the cross-sectional survey study (in German) with questions on the assessment of the current care situation, possible chronification factors, and specific therapy requirements. The survey was web-based and anonymized, but respondents could voluntarily provide information on their workplace in order to support mapping the nationwide care of patients with cancer-related pain. We distributed the survey link and QR code to German Pain Society members by circular e-mail on April 3, 2023, sent two reminders to participate (June 7, 2023, and August 21, 2023), and estimated survey completion would take circa 10 min. The data collection period was from April 3, 2023, to September 30, 2023. The survey was conducted in accordance with the Declaration of Helsinki. As this study was a survey of healthcare professionals with voluntary participation, ethics approval was waived by the ethics committee of the University of Ulm.

### Participants

All German Pain Society members received a link to take part in the online survey. Due to the survey’s limited focus on cancer-related pain, we emphasized our particular interest in having Society members directly involved in treating patients with this clinical entity complete the survey. No sampling methods were applied. At the beginning of the survey, participants had to give their informed consent to take part in data processing.

### The survey

In detail, the survey items were used to assess care provider qualifications and the current care structure and treatment options for acute and chronic cancer-related pain at respondents’ workplaces.^1^ Furthermore, the survey queried on the subjective assessment of the quality of care (regional and nationwide in general). As a federal country, Germany has regional differences in the healthcare system with regard to medical care structures. To determine possible differences, we used the first two digits of the postal code that define the 95 postal regions. A postal region is used for the geographical allocation of mail in Germany and can include individual cities or municipalities. Several medical facilities such as hospitals can be located in the corresponding postal regions. Further on, we asked for possible specific chronification factors of cancer-related pain and ideas and suggestions for therapy concepts for chronic cancer-related pain in order to optimize the future care of this specific patient group from a pain medicine perspective. Some items were multiple choice (single answer and multiple answer questions); others offered dichotomous or 11-point NRS (numerical rating scale) or free-form response options. The assessment of the care situation using NRS was categorized as follows: NRS 0–2 = very poor, NRS > 2–4 = poor, NRS > 4–6 = fair, NRS > 6–8 = good, and NRS > 8–10 = very good. Apart from items seeking information on demographics or the current care structure, the survey aimed to obtain participants’ subjective assessments. All questions asked had to be answered (except free-form responses).

In order to assess the regional care situation, we asked participants to voluntarily indicate their postal region. They could also decide if they wanted to provide the name(s) of their department, clinic, or practice. When multiple participants from the same department were asked about the departmental structure and therapy services, only one answer was counted. Thus, this part of the results should be understood per participating department. When different information on the range of offered therapies was provided, the more comprehensive answer was counted. The categorization of the free text information was carried out by the authors through a multi-stage procedure.

All survey items were developed independently for the study using the Delphi method in a multi-stage process. An interdisciplinary team of doctors and psychotherapists from the German Pain Society’s Cancer Pain Working Group was involved in the design.^2^ The questionnaire was not validated.

### Statistical analysis

The frequency distributions were presented using absolute frequency data (*n*), percentages (*%*), and medians (*Mdn, Q1-Q3*). As most of the variables used to assess the care situation were at the ordinal data level and not normally distributed, the asymptotic Wilcoxon test was used to analyze differences. When assessing the regional care situation for cancer-related pain, the median of the responses was formed for several participants from a postal region. The significance level was set to *α* = 0.05. All statistical analyses were performed with SPSS Statistics Version28 (IBM Corporation, Armonk, NY, USA).

## Results

### Participants

We received and analyzed data from 159 German Pain Society members (4.27*%* of 3722 members in October 2023). Altogether, 237 members accessed the survey. Of them, 22.8% (*n* = 54) terminated their completion at the section with cancer-related pain questions at the beginning of the survey, and these data sets were not analyzed.

Participants were primarily physicians (*n* = 148, 93.1*%*), with anesthesiologists (75.7*%)* comprising the largest specialty area (*n* = 112, *n* = 22 missing value). Participating physicians also had varying additional qualifications: 83.8*%* (*n* = 124) as pain therapy specialists and 57.4*%* (*n* = 85) in palliative medicine, whereas 55.4% (*n* = 82) had both of these qualifications. We received fewer responses from other professions: nursing (*n* = 5, 3.1*%*), psychotherapy (*n* = 4, 2.5*%*), physiotherapy (*n* = 1, 0.6*%*), and occupational therapy (*n* = 1, 0.6*%*), who all had additional specialty qualifications in pain treatment. In terms of professional experience in pain therapy, 74.2*%* (*n* = 118) reported having + 6 years of experience, with 10.1% (*n* = 16) having 3–5 years and 5.7% (*n* = 9*; n* = 16 missing value) 0–2 years. Consequently, the participants were a highly qualified group of pain therapy experts.

A large majority of participants work in hospitals (71.0*%, n* = 113), with fewer, 37.7*%* (*n* = 60) in forms of outpatient care (e.g., private office, healthcare centers), and a small portion exclusively in specialized outpatient palliative care (1.8*%, n* = 3) or non-clinical care facilities (0.6*%, n* = 1). Some participants reported working at multiple sites (13.2%, *n* = 21).

Respondents reported that a smaller proportion of their care work comprised treating people with cancer-related pain, with 76.7% (*n* = 122) saying less than 25% of their patients have this distinct clinical entity. A further 18.9% (*n* = 30) reported between 25 and 50% of their patients suffer from cancer-related pain. Only 4.4% (*n* = 7) reported more than 50% of their care work involved patients experiencing cancer-related pain.

### Current care situation

#### Assessment of the quality of care for acute and chronic cancer-related pain

**Fig. 1:**
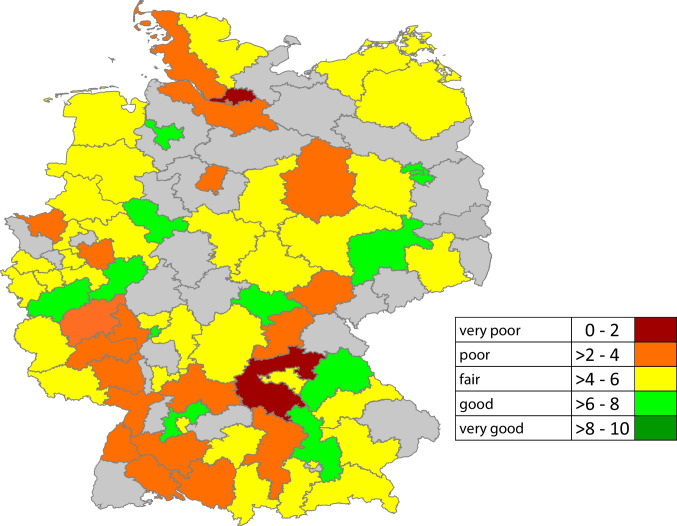
Assessment of the regional care situation for chronic cancer-related pain based on Germany’s postal regions (*n* = 69 of the 95 postal regions; responses per postal region: 28 postal regions with 1, 19 with 2, 9 with 3, 10 with 4, 2 with 5, and 1 with 7 responses). If there were several responses from one postal region, the median was formed; fields shaded in gray = no response from the postal region code; six participants did not specify their postal region and are not shown

Participants were asked to rate the care of chronic cancer-related pain regionally and nationwide. Figure 1 presents the at times significant differences in the assessment of the regional care of chronic cancer-related pain. Between one and seven assignable responses per postal region were collected. In some cases, very different assessments were given by the individual respondents within a postal region.

Overall, assessment of care for chronic cancer-related pain at the regional level was better (*Mdn* 5, Q1–Q3 3–7; NRS 0 = very poor, 10 = very good) than care provided at the Germany-wide level ((*Mdn* 4, Q1–Q3 3–6; *asymptomatic Wilcoxon test*: *z* = − 4.59, *p* < 0.001, *n* = 148). In contrast, the Germany-wide assessment of care for acute cancer-related pain was more positive (*Mdn* 5, Q1–Q3 4–6) than that of chronic cancer-related pain (*asymptomatic Wilcoxon test*: *z* = − 2.00, *p* = 0.046, *n* = 148). There was no difference between the assessments of regional care for acute (*Mdn* 6, Q1–Q3 3–7) and chronic cancer-related pain (*asymptomatic Wilcoxon test*: *z* = − 1.91, *p* = 0.056, *n* = 148).

#### Care options for chronic cancer-related pain

When asked about available care services at their workplace, participants from 91 departments (58.7*%*) reported at least one interdisciplinary multimodal treatment option for patients with chronic cancer-related pain within the same IMPT approach as patients with chronic non-cancer pain. In 38.1*%* (*n* = 59), no such treatment option was available. Most interdisciplinary pain therapy options were provided on an outpatient basis (39.0*%*, *n* = 62), with a smaller number offering therapy at a day clinic (11.6*%*, *n* = 18) and/or inpatient IMPT (29.6*%*, *n* = 47) (excluded cases: *n* = 5 missing values, *n* = 4 excluded cases due to duplicated information from the same department, *n* = 1 option not known).

Over 80*%* (80.5*%*, *n* = 128) of sites had no specific treatment offered for chronic cancer-related pain, while 10% (*n* = 16) offered such treatment. Six participants (3.7*%*) reported they did not know (missing values: *n* = 5, excluded cases due to duplicated information from the same department: *n* = 4).

**Fig. 2:**
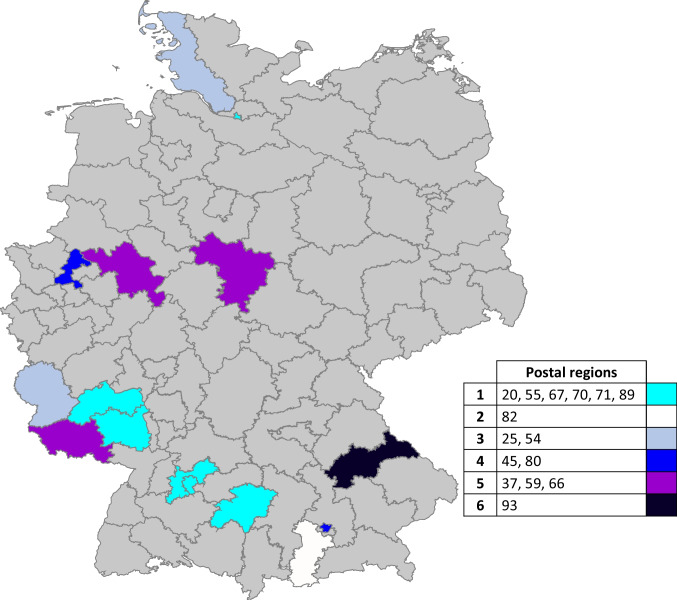
Distribution of facilities with specific therapy services for patients with chronic cancer-related pain (*n* = 15) based on Germany’s postal regions: 1 = outpatient only, 2 = day clinic only, 3 = inpatient only, 4 = outpatient + day clinic, 5 = outpatient + inpatient, 6 = outpatient, day clinic, and inpatient (gray shaded fields = no response from the postal region). A participant with a specific therapy offer available at the facility (outpatient and day clinic) has no indication of the postal region and is not shown

The specific outpatient options were dominated by consultations with medical (*n* = 11) and psycho-oncology specialists (*n* = 7). Pain-specific psychotherapeutic (*n* = 3) or other therapies (*n* = 1) were the exceptions. Options for specific IMPT at a day clinic, inpatient or interdisciplinary, outpatient treatment procedures were relatively evenly spread: day clinic (*n* = 5), inpatient (*n* = 6), and outpatient (*n* = 7). For a regional overview of specialty care options for chronic cancer-related pain, see Fig. 2.

### Assessment of chronification mechanisms in cancer-related pain from the participants’ perspective

More than 2/3 of the experts surveyed from the field of pain medicine assumed special chronification mechanisms for cancer-related pain (67.9*%, n* = 108), while 18.2*%* (*n* = 29) did not assume this and 5*%* (*n* = 8) were unable to assess it (*n* = 14 missing values).

Participants identified psychological influencing factors involving cancer-related or general fears. In the medical context, they primarily mentioned therapy-related causes, but also deficits in the care situation and physicians’ knowledge as possible pain chronification factors. From a sociomedical point of view, participants identified negatively altered situations in their professional and private environment as relevant influencing factors (Table 1*)*.

**Table 1:** Reasons participants reported for the chronification of cancer-related pain (responses were received from 108 participants, most of whom gave multiple answers)

Dimensions	Chronification factors	Number of mentions
Psychological dimension	Psychological. general factors (e.g., ability to adapt, psychological comorbidities)	32
	Specific anxieties (e.g., fear of progression, recurrence, death)	25
	Anxiety in general	20
	Existential illness/loss of control	16
	Depression	11
	Grief/parting/acceptance	7
Medical dimension (including the care situation)	Therapy-related causes (e.g., polyneuropathy, fatigue)	32
	Cancer-/tumor-related factors (e.g., neuropathic pain, inflammatory components)	23
	General deficits in care (e.g., too few specialized therapies)	22
	Insufficient knowledge/expertise among practitioners/inadequate therapy (e.g., lack of pain therapy expertise, inadequate drug treatment)	17
	Insufficient knowledge/refusal to provide information/lack of acceptance of psychological cofactors by the patient	4
	Palliative care aspects (e.g., total pain, tumor progression)	4
Sociomedical dimension	Social factors (e.g., loss of roll, isolation)	21
	Psychosocial factors in general	11

### Assessment of the need for specialized care for chronic cancer-related pain

**Fig. 3:**
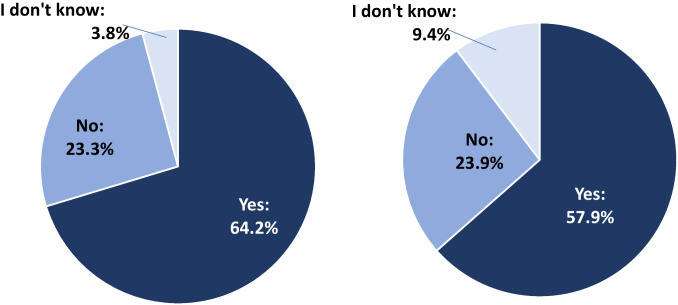
Assessment of the need for specific therapy concepts (left) and an IMPT approach (right) as a therapy concept for chronic cancer-related pain (proportion of participants (%); left: *n* = 14 missing values for *n* = 159 participants; right: *n* = 11 missing values for *n* = 159 participants)

Whereas at present, there is a low availability of specific therapy options for patients with chronic cancer-related pain, the majority of participants saw a need for such a therapy concept. More than half considered IMPT to be a suitable fundamental approach (Fig. 3).

Besides the pain-focused psychotherapy, participants recommended that specific psycho-oncological therapy elements should preferably be integrated in the IMPT context for patients suffering from chronic cancer-related pain (Table 2).

**Table 2:** Specific therapy elements named as necessary/required for chronic cancer-related pain. Responses were received from 87 participants, most of whom gave multiple answers

Specific therapy element	Number of mentions
Psychotherapy targeted to cancer-related pain	64
Inclusion of special therapies	32
Pain medication treatment	26
Strengthening interdisciplinary, multi-professional Cooperation	19
Improving organizational processes	18
Dealing with cancer-related restrictions	18
Including social environment/social counselling	12
Spirituality	11
Oncological therapy/palliative medicine	6
Specific group therapy options	4

In addition to expertise in pain medicine, 69.8*%* (*n* = 111) of participants considered specific expertise in treating cancer-related pain as necessary, while 17.0% (*n* = 27) saw no need for this. Additional expertise was considered equally important for all professions. In the free-form responses on possibilities for improvement, generally better expertise, networking, and interdisciplinary cooperation were primarily mentioned (Table 3).

**Table 3:** Free-form responses on improving care of patients with chronic cancer-related pain (by thematic topics; responses from 33 participants are available, the majority with several free-form entries)

Thematic topics	Number of mentions
Specialty training and professional development/improved expertise	14
Cooperation/interdisciplinarity/strengthening care structures	14
Improve funding/framework conditions	7
Overcoming knowledge deficits on chronic cancer-related pain	5
Patient education/information	5
Providing targeted pain therapy options including IMPT	4
More frequent integration of pain medicine and transfer of expertise	3
More critical and rational usage of medications	3

## Discussion

Currently, the number of long-term cancer survivors is increasing significantly and will continue to do so in the future. Chronic cancer-related pain is common in this group and leads to a high psychosocial burden. Our survey of members of the German Pain Society, a group with particular pain therapy expertise in treating this distinct clinical entity, reveals considerable care deficits. Specific chronification factors were assumed to be at the psychic, medical, and social levels, with psychological factors such as cancer-related anxiety playing a particularly important role [7, 17]. Participants considered a specific range of therapies and specialist expertise on the part of the practitioners as necessary for this distinct clinical entity.

### Care situation for cancer-related pain

Participants assessed the regional care of chronic cancer-related pain very differently, in some cases even within a postal region. One-third rated the care situation in their region as poor. Across Germany, the care situation for acute cancer-related pain was assessed as better than that of chronic cancer-related pain. A possible explanation for this difference might be that there are numerous guideline recommendations for the treatment of acute cancer-related pain [18, 19]. In contrast, chronic cancer-related pain is not explicitly named as such in the existing German guidelines [20, 21]. While international guidelines do exist, they focus much more on pharmacological interventions and an integrative multimodal therapeutic approach plays a subordinated role [16, 22, 23]. A stage-appropriate differentiation of cancer-related pain, as has already been published and repeatedly called for [24, 25], has yet to be established. Thus, pain chronification and chronically persistent cancer-related pain are not sufficiently recognized by practitioners, patients, or the general public in terms of the chronification mechanisms and their biopsychosocial, therapy-relevant characteristics. Hence, this group of patients has to date been inadequately treated and underserved.

IMPT is a long-established and effective pain management approach for chronic pain syndromes [12, 13]. The biomedical, psychological, and social factors that influence chronic pain are treated in an interprofessional setting over 3–4 weeks. The team-integrated multimodal approach and the close involvement of patients in individual and group settings had positive effects on each pain-influencing factor. This approach is increasingly being recommended for chronic cancer-related pain [16, 22, 23, 26]. Overall, the participants indicated a specific, mostly outpatient (e.g., in the form of special consultation hours) therapy provision for cancer-related pain at only 16 (10%) of the facilities and a specific day clinic or inpatient IMPT provision at only 10 facilities (6.3%). In contrast, the German Federal Joint Committee^3^ identified 445 hospitals with inpatient or day clinic IMPT services in Germany in 2018, but had already assumed there was a general care deficit in the area of non-cancer pain [27, 28]. Furthermore, the German health system does not provide an independent funding framework for outpatient IMPT services outside of current research projects [29, 30]. The information on outpatient services is therefore presumably based on informal cooperation. However, this does not meet the requirements of IMPT, which include a formalized, team-integrated approach [12]. Overall, there is a considerable deficit in specific and comprehensive interdisciplinary multimodal care for the increasing number of long-term survivors with chronic cancer-related pain.

### Chronification factors in cancer-related pain

The biopsychosocial pain model is considered the basis for the chronification and persistence of pain [31]. The participants of the present survey named cancer-specific chronification factors at all levels. The most frequently mentioned problem was psychological factors, in particular specific fears such as fear of recurrence or fear of death. These factors and their interaction with the perception of pain are also discussed in detail in the German guideline on psycho-oncology [32] although not explicitly as potential factors with regard to pain chronification. Initial cross-sectional studies were able to identify anxiety as a risk factor for cancer-related pain chronification [7, 33, 34] and insufficient expertise of the treating physicians with regard to chronic cancer-related pain, including uncritical use of opioids and especially prolonged opioid use in long-term survivors [35]. The need for improved networking and interdisciplinary cooperation has long been identified [36].

### Specific therapeutic approaches for chronic cancer-related pain

Patients with chronic cancer-related pain predominantly receive care within the same IMPT approach being taken with patients suffering from non-cancer pain. However, the majority of experts see a great need for specific and separate treatment approaches, including cancer-specific IMPT. Cancer-related pain psychotherapy was named the most important therapy element. Survey participants were predominantly physicians, over 50% of whom were qualified in palliative medicine and therefore aware of the psychological and spiritual components of Dame Cicely Saunders’ “total pain” concept [37]. Although these two components are perceived as essential elements in psycho-oncological therapy, the participants saw a considerable need for improvement in the area of chronic cancer-related pain. Furthermore, by definition, palliative care cannot refer to the target group of long-term survivors our study addressed [20].

The low proportion of participants from the field of psychotherapy could indicate a low representation of this professional group in the care of cancer-related pain. In this context, it is significant that in Germany, cancer-specific aspects are not adequately taught in the advanced training curriculum “Special Pain Psychotherapy”, whereas the German Cancer Society’s advanced training curriculum “Psycho-oncology” does not adequately teach the distinct clinical entity of “chronic cancer-related pain”.

The majority of participants emphasized the need for special expertise from all professional groups involved in order to do justice to the complexity of the distinct clinical entity, chronic cancer-related pain.

### Optimization potential

The fundamental basis for optimizing the care situation should be raising awareness of the problem of chronic cancer-related pain and healthcare professional education at all levels and for all professions.

Using new media such as online platforms, virtual reality, telemedicine, or smartphone applications could contribute to improving the care of chronic cancer-related pain [38]. The development of specific tools for the early identification of patients at risk of pain chronification and timely access to targeted therapy is particularly necessary given the increasing number of long-term survivors [5]. Sociomedical stress factors such as limited ability to work [39] and the resulting financial burdens and restrictions on social participation must be identified and adequately taken into account within the comprehensive care of this distinct clinical population, e.g., within an IMPT treatment approach.

Expanding special consultation hours and closer networking of oncology professionals with the involvement of pain experts, for example by establishing interdisciplinary pain boards—comparable to tumor boards—would point the way forward. Clarifying who is responsible for this special group of patients (GP, specialist or oncologist, pain physician, palliative physician) would also contribute to improved care, whereby the assignment should be individualized and adapted to the disease stage.

### Limitations and strengths

A relatively small number of German Pain Society members participated in the study, perhaps because few members are involved in the regular care of the patient group the survey targeted. For this reason, the survey cannot be representative of the members of the German Pain Society. That the majority of survey terminations occurred at the first question on patients with cancer-related pain supports this assumption. However, this limitation also underlines the existing care deficit. The extent to which the strong focus on physicians with little interdisciplinary participation impacted the results cannot be ascertained.

Respondents were characterized by years of expertise and additional qualifications and represented over 70% of the postal code regions. Additionally, the assessment of regional care could also refer to areas outside of a participant’s own postal region.

The survey was conducted among members of the German Pain Society, as they have particular expertise in the treatment of patients with chronic pain with regard to the biopsychosocial pain model and the resulting therapy concepts, including IMPT. However, other groups such as oncologists or palliative care physicians also treat patients with cancer pain. They may assess the care situation and the needs of those affected differently.

The data is therefore not representative of the individual regions or of Germany as a whole and cannot be generalized—let alone transferred to other countries. Nevertheless, we believe that a good overview of the care of chronic cancer-related pain has been obtained and, as the first study of its kind, it provides the basis for further research and planning to improve the care situation.

Due to a lack of a comparable questionnaire, this one was created by the authors and is therefore not validated. In this context, however, it should be noted that these are mainly descriptive analyses.

Finally, we cannot rule out a response bias with regard to an increased number of participants working at facilities where such patients are treated and special therapy approaches exist. If this were the case, though, the data collected would tend to be better than it actually is.

## Conclusions

In this cross-sectional study, pain experts of the German Pain Society assume that there is a relevant care deficit in Germany for long-time survivors with chronic cancer-related pain, who are often suffering from considerable psychic distress [40]. While the survey focused on the care situation in Germany, the authors think the results widely can be transferred to other countries and therefore demonstrate the urgency for optimization. Jacobs and Shulman [36] outlined the need for developing measures to improve care for long-term survivors. However, cancer-related chronic pain is not explicitly mentioned, but at most implied in the area of tertiary prevention. The US guidelines [16, 22, 23] and other actual publications [24] acknowledge the existence of relevant psychological factors in chronic cancer-related pain, but still focus predominantly on pharmacologic interventions. In contrast, an interdisciplinary multimodal approach to meet the complex needs to treat this distinct clinical entity is hardly considered in these recommendations. There is a need to develop comprehensive, specific therapy elements for patients with chronic cancer-related pain. In addition to a general expansion of care provision, more expertise is required with regard to therapies for this distinct clinical entity.

Therefore, future research should aim to develop cancer-specific IMPT [36]. Further clarification is needed on (1) the extent this should happen in distinct group settings only for patients with cancer-related pain or jointly with patients with non-cancer pain and (2) the extent further aspects such as spirituality should be included [41, 42]. Interviews with patients can help more clearly identify deficits and needs and develop therapy concepts with patient representatives, such as specific psychotherapeutic interventions, including group manuals. Finally, more research is needed on the sustainability of such therapeutic measures, particularly with regard to longer-term effectiveness [43, 44].

## Supplementary Information

Below is the link to the electronic supplementary material.

Supplementary file1 (DOCX 144 KB)

## Data Availability

Data is provided as Excel file upon request from the corresponding author.

## Footnotes

1 See the supplementary for an English translation of the survey.

2 Note: The authors are members of the German Pain Society but did not take part in the survey.

3 The Federal Joint Committee (*Gemeinsamer Bundesausschuss*) is a public legal body made up of the four leading umbrella organizations of Germany ‘s self-governing healthcare system.
