# Supplementary material for: Cancer-related pain in long-term survivors of oncological diseases: results of a survey on the current care situation
Source: Support Care Cancer. 2024 Dec 20;33(1):44. doi: 10.1007/s00520-024-09081-2 (PMC11662055; doi:10.1007/s00520-024-09081-2)
Supplement: Supplementary file 1 — Supplementary file1 (DOCX 144 KB) [file 520_2024_9081_MOESM1_ESM.docx]

*Please note: This is an English translation of the online survey which was in the German language. Additionally, the original online survey made use of conditional branching/skip logic so that respondents saw some questions (or not) based on their answers to previous questions. Here, we use an * to indicate a question presented only to participants to whom it applied as revealed by their responses to earlier survey items.*

**Dear colleagues,**

**Pain can significantly impact the life quality of patients with cancer. There are established treatment concepts for acute cancer-related pain, e.g. in the context of initial diagnosis or tumor progression. Fortunately, life expectancy after or with cancer continues to increase due to ever-improving treatment options. As the number of long-term survivors increases, so does the number of patients with chronic cancer-related pain. This study aims to assess the current care situation for patients with cancer-related pain in Germany and derive possible potential for improvement.**

**We would be most appreciative if you would take about 10 minutes to answer the online survey and thus contribute to improving the care of patients with cancer-related pain.**

**Thank you in advance for your participation!**

**Yours sincerely,**

**H. Hofbauer, S. Wirz, U. Stamer, A. Bundscherer, F. Rapp, K. Kieselbach**

**Further information:**

**This survey is an initiative of the German Pain Society's Cancer Pain Working Group.**

**If you have any questions, comments or suggestions, please send them by e-mail to: hannes.hofbauer@uni-ulm.de**

**These first questions ask you to provide information about your professional area and the care structure of the facility where you work and its postal/ZIP code.**

Which professional area do you work in?


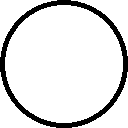
 Medicine (physicians)

| 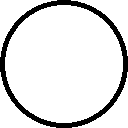 Occupational therapy |
| --- |


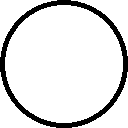
 Nursing

| 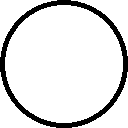 Physical therapy |
| --- |


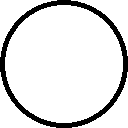
 Psychotherapy

| 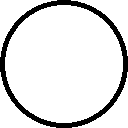 Other health care field/area:  __________________________________ |
| --- |

Please share in what type of health care facility/structure you work in.


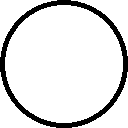
 University hospital

| 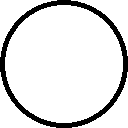 Maximum care hospital |
| --- |


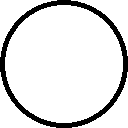
 Hospital for specialized care

| 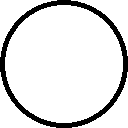 Hospital for basic and standard care |
| --- |


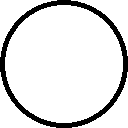
 Physician’s office/health care center

| 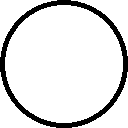 Other health care facility/structure (please specify)  __________________________________ |
| --- |

We would also like to record regional differences in care. Please enter the **postal code** (minimum first 2 digits) of your clinic/physician’s office/health care center, etc.! **_________________**

**In the following, we ask you to answer questions about patients with cancer-related pain and the corresponding treatment options in your care unit.**

**In this context, care unit refers to your entire clinic, physician's practice or health care center, even if you may not personally work in individual therapy services. If you cannot provide an exact number, please provide an estimate if possible!**

**Please note that questions about treatment options for patients with cancer-related pain refer to treatment options specifically tailored to this group, which also take into account the special features of pain in the context of cancer/tumor diseases.**

How many patients with pain (non-cancer and cancer-related) do you treat in your care unit each year?

**________** per year

What estimated proportion of patients do you treat for **cancer-related pain**?

Proportion of patients with acute cancer pain (e.g., due to tumor progression, metastasis, etc.):

**__________**

Proportion of patients with chronic cancer pain (e.g., curatively treated, in remission for at least one year, in a stable palliative situation for at least one year.)

**__________**

Does your care unit offer a special outpatient therapy program that is **only** tailored to patients with **acute**, cancer-related pain?


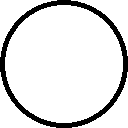
 Yes

| 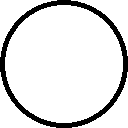 No |
| --- |


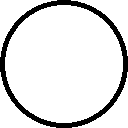
 I don’t know.

If yes, what type of outpatient therapy is offered?


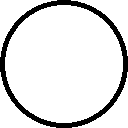
 Medical treatment (by physicians)

| 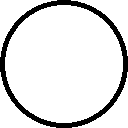 (Pain) psychotherapy |
| --- |


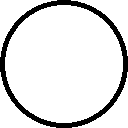
 Psycho-oncology

| 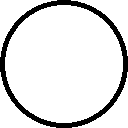 Other (please specify) ___________________ |
| --- |

In your care unit, do you treat patients with **chronic cancer-related** pain together with patients with **non-cancer** pain as part of interdisciplinary, multimodal pain therapy?


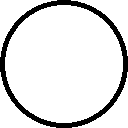
 Yes, outpatient

| 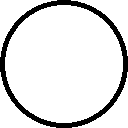 Yes, day clinic |
| --- |


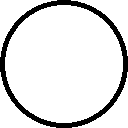
 Yes, inpatient

| 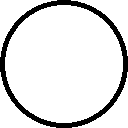 No |
| --- |


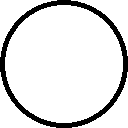
 I don’t know

Does your care unit offer a special range of therapies that are **only** tailored to patients with **chronic** cancer-related pain?


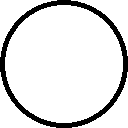
 Yes

| 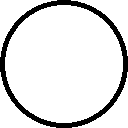 No |
| --- |


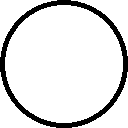
 I don’t know.

If yes, which type of therapy(ies)?


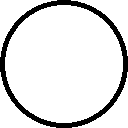
 Consultation with a physician

| 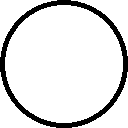 Specialist (pain) psychotherapy consultation |
| --- |


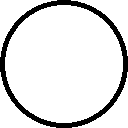
 Special psycho-oncological consultation

| 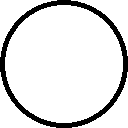 Other types of outpatient therapy (please specify) **_______________** |
| --- |


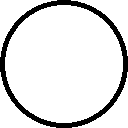
 Outpatient

| 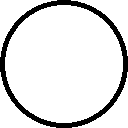 Day clinic |
| --- |


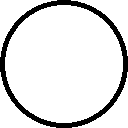
 Inpatient

| 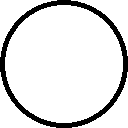 Other therapy(ies) (Please specify) **_____________** |
| --- |

Do you yourself or in cooperation offer invasive pain therapy techniques/measures for patients with cancer-related pain?


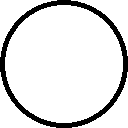
 Yes

| 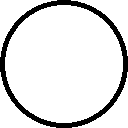 No |
| --- |


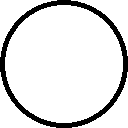
 I don’t know.

If yes, what type(s)?


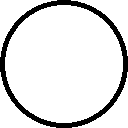
 Implantation of intrathecal pump systems

| 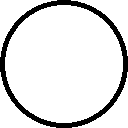 Implantation of intrathecal port systems |
| --- |


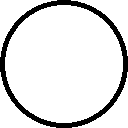
 Insertion of peridural catheters with external pump supply

| 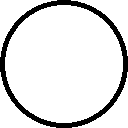 Placement of peripheral regional anesthesia catheters |
| --- |


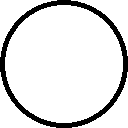
 Ablative/neurodestructive procedures in the spinal cord area

| 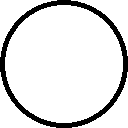 Coeliac plexus neurolysis |
| --- |


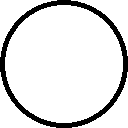
 Implantation of stimulation systems (e.g., SCS probes)

| 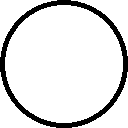 Others (please specify!) **_________________________________________** |
| --- |

**In the following, we ask you to give us your assessment of pain therapy care for patients with cancer-related pain. The next questions relate to the care situation for acute cancer-related pain.**

How would you rate the **general** care services **in Germany** are for patients with **acute** cancer-related pain?


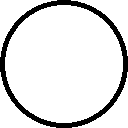


Very poor


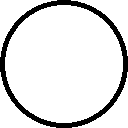


-4


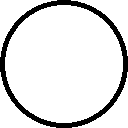


-3


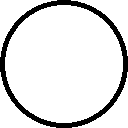


-2


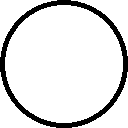


-1


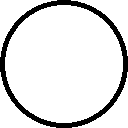


0


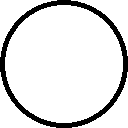


+1


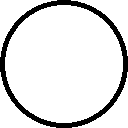


+2


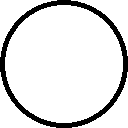


+3


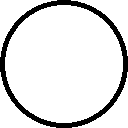


+4


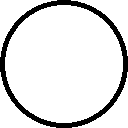


Very good

How would you rate the care services **in your region** are for patients with **acute** cancer-related pain?


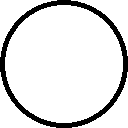


Very poor


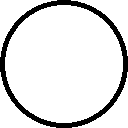


-4


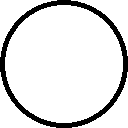


-3


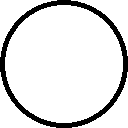


-2


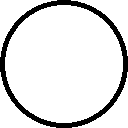


-1


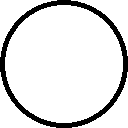


0


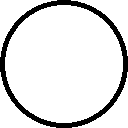


+1


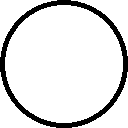


+2


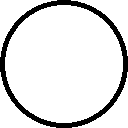


+3


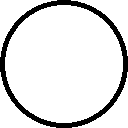


+4


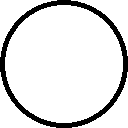


Very good

For which reasons do you suppose there is a lack of care for patients with **acute** cancer-related pain **in your region**?


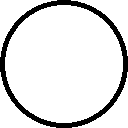
 Patient inquiries

| 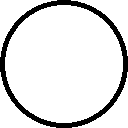 Feedback from health care professionals |
| --- |


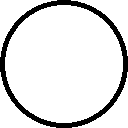
 Personal opinion

| 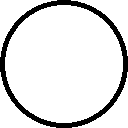 Other:  _______________________________________________ |
| --- |

**The next questions concern the care situation for chronic cancer-related pain.**

How would you rate the **general** care services **in Germany** are for patients with **chronic** cancer-associated pain?


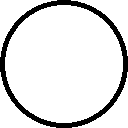


Very poor


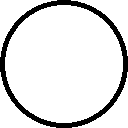


-4


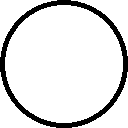


-3


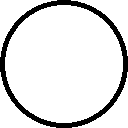


-2


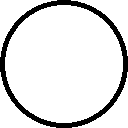


-1


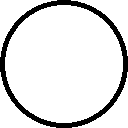


0


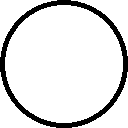


+1


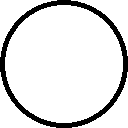


+2


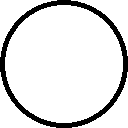


+3


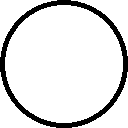


+4


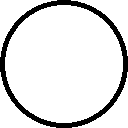


Very good

How would you rate the care services in **your region** are for patients with **chronic** cancer-related pain?


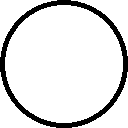


Very poor


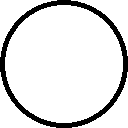


-4


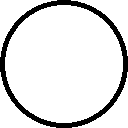


-3


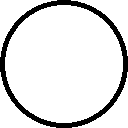


-2


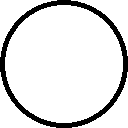


-1


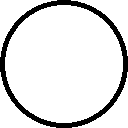


0


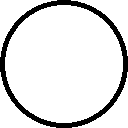


+1


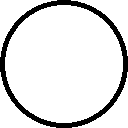


+2


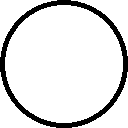


+3


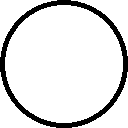


+4


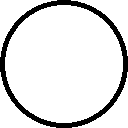


Very good

Why do you suppose there is a lack of care for patients with **chronic** cancer-related pain **in your region**?


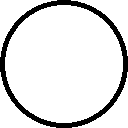
 Patient inquiries

| 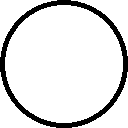 Feedback from health care professionals |
| --- |


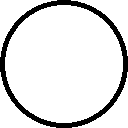
 Personal opinion

| 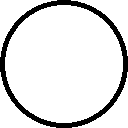 Others: **_______________** |
| --- |

Do you think that different/additional factors play a role in the **chronification** of **cancer-related** pain compared to pain that is **not cancer-related**?


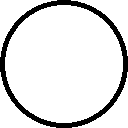
 Yes

| 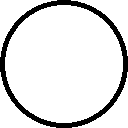 No |
| --- |

I cannot assess this.

What specific **chronification factors** do you consider important in **cancer-related pain**?

1. **_________________________**

2. **_________________________**

3. **_________________________**

Do you think that more specific expertise beyond pain medicine is needed for the treatment of patients with **chronic cancer-related** pain?

Yes

| No |
| --- |

I cannot assess this.

For which professional groups is expertise beyond pain medicine necessary for patients with **chronic cancer-related** pain?

Physicians

| Occupational therapists |
| --- |

Nurses

| Physiotherapists |
| --- |

Psychotherapists

Other health care professionals (please specify):

**________________________________________________________**

Do you think that patients with **chronic cancer-related pain** need a different treatment concept than patients with chronic **non-cancer pain**?

Yes

| No |
| --- |

I cannot assess this.

Do you think that a **specific interdisciplinary, multimodal pain therapy approach** for patients with **chronic cancer-related pain** is needed?

Yes

| No |
| --- |

I cannot assess this.

In your opinion, what specific therapy elements are necessary in such a setting in order to take into account the special characteristics of patients with chronic cancer-related pain?

1. **______________________**

2. **______________________**

3. **______________________**

**In the last part of the survey, we would like to ask you more about your qualifications and your workplace.***

Please indicate your medical specialty.

General Medicine

| Anesthesiology |
| --- |

Internal medicine

| Internal medicine and Hematology and Oncology |
| --- |

Neurosurgery

| Neurology |
| --- |

Orthopedics

| Psychiatry and Psychotherapy |
| --- |

Psychosomatic Medicine and Psychotherapy

| Trauma Surgery |
| --- |

Other ________________________________

| If currently doing specialty training, which specialty area? **______________________** |
| --- |

If applicable, please indicate your additional medical qualification.*

Special pain therapy

| Palliative medicine |
| --- |

Medical psychotherapy

| Other **______________________** |
| --- |

No additional qualifications

**In the final section if the survey, we would like to ask you more about your qualifications and your workplace.***

Are you a licensed psychologist or psychotherapist?

Yes

| No, but in professional training to become a psychologist/psychotherapist |
| --- |

No, and also not in professional training to become one

Have you earned additional professional qualifications?*

Specialized pain psychotherapy

| Psycho-oncology |
| --- |

Other

**Now, we would like to ask you in more detail about your qualifications and your workplace.***

Do you have specialized training in the field of pain medicine (e.g., pain physiotherapy, pain nurse/algesiological specialist assistant, specialized pain nursing/care giving ...)?

Yes

| No |
| --- |

**Do you work full-time or part-time?**

Full-time (100%)

| Part-time, please specify with the percentage **___________** |
| --- |

What percentage of your work time do you spend treating patients with pain disorders?

Percent **______________**

How many years of professional experience do you have in the field of **pain medicine**?

0-2 Years

| 3-5 Years |
| --- |

≥ 6 Years

Please indicate in which **hospital/clinic** department or unit you work in?*

Pain outpatient clinic

| Outpatient interdisciplinary pain therapy |
| --- |

Day hospital/clinic interdisciplinary pain therapy

| Inpatient interdisciplinary pain therapy |
| --- |

Palliative care unit

| Oncology outpatient clinic, day hospital, ward |
| --- |

Another hospital/clinic department/unit (please specify):

Does your **hospital**/**clinic** offer interdisciplinary, multimodal pain therapy?

Outpatient

| Day hospital/clinic |
| --- |

Inpatient

| I don’t know. |
| --- |

Does your care unit (physician’s **office, health care center, other care structure**) offer or participate in **outpatient, interdisciplinary, multimodal pain therapy**?

Yes

| No |
| --- |

I don’t know.

**Finally, we have one more request:**

We would like to create a "map" of available therapies for patients with cancer-related pain. For this purpose, we would ask you to indicate your exact place of work (including the department in the case of hospitals/clinics).

**Thank you very much!**

Hospital or clinic/health care center/physician’s office

Department/unit (if applicable) **_______________________________**

Street **_______________________________**

Number **_______________________________**

Postal/ZIP code **_______________________________**

City **_______________________________**

Telephone number **_______________________________**

E-mail address **_______________________________**

Do you have any general comments or ideas on how the care of patients with cancer-related pain can be improved?

**________________________________________________________________________________________________________________________________________________________________________________________________________________________________________________**

**Please conclude the survey by pressing the "CONTINUE" button!**

**Thank you very much for participating and helping us to improve the care situation of patients with cancer-related pain!**
